# Genome Size and Labellum Epidermal Cell Size Are Evolutionarily Correlated With Floral Longevity in *Paphiopedilum* Species

**DOI:** 10.3389/fpls.2021.793516

**Published:** 2021-12-16

**Authors:** Feng-Ping Zhang, Shi-Bao Zhang

**Affiliations:** ^1^College of Traditional Chinese Medicine, Yunnan University of Chinese Medicine, Kunming, China; ^2^Key Laboratory of Economic Plants and Biotechnology, Yunnan Key Laboratory for Wild Plant Resources, Kunming Institute of Botany, Chinese Academy of Sciences, Kunming, China

**Keywords:** genome size, floral longevity, cell size, flower, leaf, slipper orchids

## Abstract

Genome size is known to influence phenotypic traits in leaves and seeds. Although genome size is closely related to cellular and developmental traits across biological kingdoms, floral longevity is a floral trait with important fitness consequence, but less is known about the link between floral longevity and sizes of genomes and cells. In this study, we examined evolutionary coordination between genome size, floral longevity, and epidermal cell size in flowers and leaves in 13 *Paphiopedilum* species. We found that, across all the study species, the genome size was positively correlated with floral longevity but negatively associated with labellum epidermal cell size, and a negative relationship was found between floral longevity and labellum epidermal cell size. This suggested that genome size is potentially correlated with floral longevity, and genome size has an important impact on life-history trait. In addition, genome size was positively correlated with leaf epidermal cell size, which was different from the relationship in flower due to different selective pressures they experienced or different functions they performed. Therefore, genome size constraints floral longevity, and it is a strong predictor of cell size. The impact of genome size on reproduction might have more implications for the evolution of flowering plants and pollination ecology.

## Introduction

It is well known that genome size varies significantly within plants, even in very closely related plant species (Šmarda and Bureš, 2010; Pellicer et al., 2018). However, the ecological adaptation and evolutionary significance of genome size remains unclear. Phylogenetic studies have revealed that increases and decreases of genome size have taken place many times within lineages (Soltis et al., 2003; Leitch et al., 2005; Simonin and Roddy, 2018). Genome size variation may suffer from strong selection pressures (Petrov, 2002), which suggests that some cost may exist to associate large genomes, or benefits from small genome size (Knight et al., 2005; Beaulieu et al., 2007). Therefore, there has been a growing interest in identifying the phenotypic consequences from species with different genome sizes (Knight et al., 2005; Beaulieu et al., 2007; Simonin and Roddy, 2018; Théroux-Rancourt et al., 2021).

Variations in genome sizes may be closely related to cell sizes across biological kingdoms (Cavalier-Smith, 2005; Simonin and Roddy, 2018; Théroux-Rancourt et al., 2021). Such coordination is indirectly and/or directly linked with whole-organism fitness characters (Jockusch, 1997). Previous studies have found that the cell size is positively correlated with genome size (Bennett, 1972; Edwards and Endrizzi, 1975; Sugiyama, 2005). In addition, there is a positive relationship between genome size and cell cycle duration (Rees et al., 1966; Baetcke et al., 1967; Bennett et al., 1983; Lawrence, 1985). In view of these cellular associations, it is assumed that many physiological and functional traits may link with genome size.

Observations have shown that there are positive and negative relationships between genome size and the structural characteristics in leaves, such as cell size, densities of veins and stomata, stomatal size, leaf mass per unit area, and leaf specific area (Beaulieu et al., 2007; Simonin and Roddy, 2018). In addition, changes in genome size are also associated with variation in many functional physiological traits, such as maximum rate of photosynthesis, minimum generation time, seed mass, flower size, and environmental variables (Bennett, 1972; Knight and Ackerly, 2002; Knight et al., 2005; Meagher et al., 2005; Beaulieu et al., 2007; Simonin and Roddy, 2018; Roddy et al., 2020; Faizulah et al., 2021; Théroux-Rancourt et al., 2021). In turn, the variation of plant traits may impact the evolution of genome size (Simonin and Roddy, 2018; Roddy et al., 2020; Théroux-Rancourt et al., 2021). It has been proposed that genome size is a predictor of cell sizes and photosynthetic rates in terrestrial vascular plants (Roddy et al., 2020). In addition, seed mass is strongly positively correlated with genome size (Beaulieu et al., 2008). Environmentally, genome size has been reported to vary with latitude, precipitation, altitude, and temperature (Sims and Price, 1985; Wakamiya et al., 1993; Knight and Ackerly, 2002; Knight et al., 2005). These suggest that variations in genome size may be adaptive.

Flower is a sexual reproductive organ of flowering plants, which has an important impact on its reproduction success (Rosas-Guerrero et al., 2014). Floral longevity is the length of duration that flowers remain open and functional, which is a key floral characteristic that impacts the reproductive fitness of plants (Primack, 1985). Floral longevity varies considerably from a day or less (e.g., *Ipomoea purpurea*) up to 2 months (e.g., some species from *Orchidaceae*) among flowering plants (Kerner von Marilaum, 1895; Gori, 1983; Primack, 1985; Ashman and Schoen, 1994; Zhang et al., 2017, 2021). Some studies have indicated that the duration of the life history in leaves was correlated with genome size within vascular plants (Finch, 1990; Morgan and Westoby, 2005; Beaulieu et al., 2007, 2008; Théroux-Rancourt et al., 2021). However, a previous study found a weak positive correlation between leaf lifespan, leaf mass per area, and genome size, suggesting that among angiosperms, genome size and leaf strategy are not related (Morgan and Westoby, 2005). Variation of floral longevity may imply different ecological strategies (Zhang et al., 2021). Therefore, understanding the genetic basis of variation in floral longevity is of great value. However, the potential link between floral longevity and the sizes of genome and cell is still lacking. We speculated that genome size may be related to floral longevity through cell size effect on physiological function within floral organs. Species with larger genome and longer floral longevity might need longer developmental duration (Feng et al., 2021).

The family *Orchidaceae* is an important group of flowering plants with great values in ornamental, medical, conservation, and evolutionary research (Zhang et al., 2018). The high diversity in morphology, floral longevity, growth form, habitat, and genome size mean that orchid species exhibit various biological and ecological consequences (Cox et al., 1998; Chase, 2005; Leitch et al., 2009; Zhang et al., 2017, 2018, 2021). The genus *Paphiopedilum* species are commonly named as “Lady's or Venus's Slipper” orchids, and these species are among the most cultivated and horticulturally important plants (Liu et al., 2009; Parveen et al., 2012). This genus *Paphiopedilum* was divided into three subgenera as follows: *Parvisepalum, Brachypetalum*, and *Paphiopedilum* (Guo et al., 2015; Tsai et al., 2020). Species of this genus are characterized by their exotic flowers with a deep shoe-shaped labellum, a structure unique to orchids that is a strongly modified petal, which is the main classification characteristics and pollination channel (Liu et al., 2009), and the droop and wilting of labellum were regarded as the end of floral longevity (Sugiura et al., 2001). Their flowers have long life span, and they can remain on the plant for 62 days (Zhang et al., 2017). However, there are few studies on factors influencing floral longevity of *Paphiopedilum* species (Zhang et al., 2017, 2021). In this study, we examined the potential link between floral longevity, sizes of genomes, and cells in 13 *Paphiopedilum* species. Specifically, we hypothesized that floral longevity is positively correlated with genome size, such that species with longer floral longevity may have larger genome. We also examined whether the scaling of genome size and cell size is similar between flower and leaf, or such relationship is different as a result of distinct selective pressures they experienced.

## Materials and Methods

### Plant Materials

A total of 13 *Paphiopedilum* species from three subgenera, namely, *Parvisepalum, Brachypetalum*, and *Paphiopedilum* were used in this study (Supplementary Figure 1). These plants are grown and kept in the greenhouse at Kunming Institute of Botany, Chinese Academy of Sciences (102°41′ 25°10′ N; elevation 1,990 m). It is helpful to eliminate the effect of environmental divergence on their growth by applying similar culturing conditions. Therefore, variations may mirror the effect of a genetic consequence.

### Genome Size

Genome size (1C, pg) (the DNA content of cells in the unreplicated phase from leaf tissue) was measured by flow cytometry in Kunming Institute of Botany, Chinese Academy of Sciences. Three heart leaves of the studied plant tops of species have not been expanded, which were collected and fixed in precooled mGb dissociation solution (45 mM MgCl_2_·6H_2_O, 20 mM MOPS, 30 mM sodium citrate, 1% (W/V) PVP 40, 0.2% (v/v) Triton X-100, 10 mM Na_2_EDTA, 20μl/ml β-mercaptoethanol, pH 7.5). The tissues immediately are chopped in the buffer with a new razor blade, and then mixed the homogenate by pipetting up and down for several times. The homogenate was filtered using the 42-μm nylon mesh and incubated with DNA fluorochrome propidium iodide (PI). PI is used at 50 mg/ml simultaneously with RNase (Dolezel and Bartos, 2005; Dolezel et al., 2007; Tian et al., 2011).

The stained nuclear suspension samples were detected by BD FACScalibur^TM^ flow cytometry (Becton, Dickinson and Company, San Jose, CA 95131, USA). The cytometer was equipped with an argon ion laser operating at 488 nm. The PI fluorescence was collected using the 620 nm fluorescence-2 (FL2) filter. The sample flow rate was set at ~100 nuclei/s, and at least 6,000 nuclei were acquired for each sample. The results were analyzed using BD CellQuest^TM^ Pro software (BD, USA). The results with coefficient of variation values (CV) < 5% were considered as reliable (Supplementary Figure 2). The emitted light fluorescence intensity of PI was detected by 488 nm blue light excitation.

By observing the fluorescence peaks of PI-DNA complex of the tested sample and the internal standard (*Zea mays*, 1C = 2.41 pg; Schnable et al., 2009), the ratio of DNA content of the two individuals can be obtained, and then multiplied by the *C* value of internal standard plant, the *C* value of the tested plant can be tested and calculated by the following equation: DNA content of tested the sample = internal standard DNA content × fluorescence intensity of tested/fluorescence intensity of internal standard (Tian et al., 2011). Orchids are known to differ in cellular ploidy levels (endopolyploidy) in different tissues (Teixeira da Silva et al., 2014; Bateman et al., 2018; Li et al., 2020). In this study, to test the differences of genome size between leaves and other tissues, tissues from labellum and root of *Paphiopedilum villosum* were used to measure the genome size. We have found that values of genome sizes between leaf and labellum and root tissues are insignificantly different (Supplementary Figure 3). The measure method of genome size (1C, pg) from labellum (the even central part of labellum, Supplementary Figure 4) and root (root tip) was the same as leaf.

### Cell Size

Six labellum from newly opened flowers from six plants of each species were fixed in a formalin acetic acid-alcohol solution (37% formaldehyde, glacial acetic acid, 95% ethanol, and deionized water in a 10:5:50:35 mixture) for microscopic analysis. The even central part of labellum was cut into a section to ensure they are flattened, which was used to measure the cell size of labellum (Supplementary Figure 4), and the sampled part of labellum in all plants was the same. Six mature leaves from six different plants per species were sampled. Abaxial epidermis from the middle part of a mature leaf was coated with a thin layer without color, transparent nail polish. These films were gently torn away from the leaf surface with tweezers. These films were mounted on a microscope slide, and images were taken using the light microscope. All samples were obtained to observe epidermal cell and measured using the ImageJ software (National Institutes of Health, Bethesda, MD, USA). Epidermal cell size was subsequently calculated as suggested by Carins Murphy et al. (2016) using the following equation: Epidermal cell size = [1 – (mean stomatal size × stomatal density)]/epidermal cell density.

### Floral Longevity

The 10–20 newly emerged floral buds from different plants per species were marked. An individual flower was regarded as “opening” when the dorsal sepal upward and any floral visitor could enter labellum. A flower was identified as “wilting” when perianth was discolored or when labellum began to wilt, thereby ending its function (Sugiura et al., 2001).

### Statistical Analysis

Correlations between variables were analyzed using both Pearson's correlation and phylogenetically independent contrasts (PIC). The evolutionary correlations were tested with PIC analysis by using the “ape” package, combining molecular phylogenetic relationships (Cox et al., 1997; Guo et al., 2015). Statistical analyses were performed using R v.4.0.0 (R Core Team, 2020).

## Results

**Figure 1:**
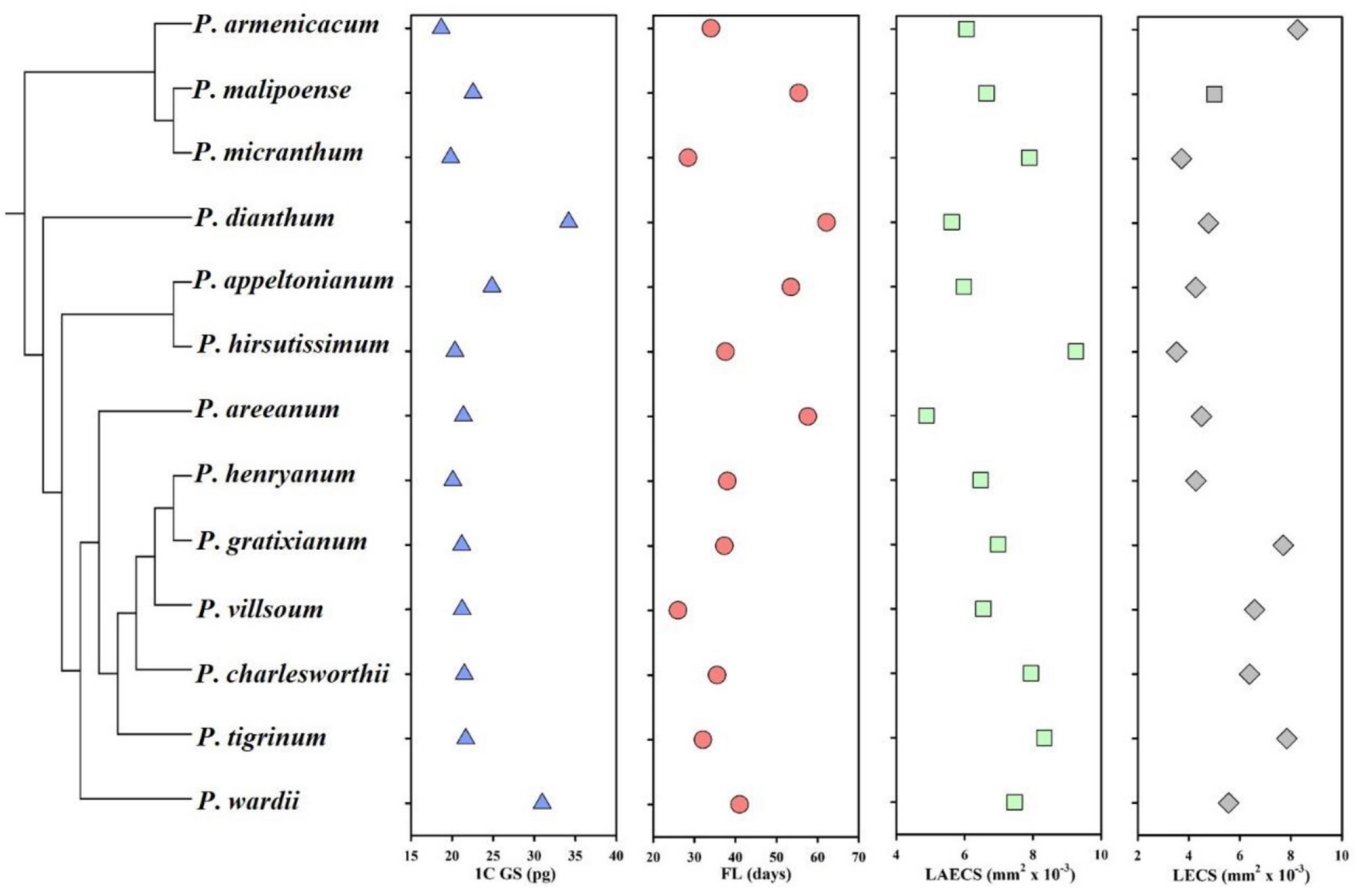
Mean of raw values in genome size, floral longevity, and epidermal cell size of labellum and leaf from 13 *Paphiopedilum* species. GS, genome size; FL, floral longevity; LAECS, labellum epidermal cell size; LECS, leaf epidermal cell size. The phylogenetic relationships of the studied *Paphiopedilum* species in this study are obtained from the study by Cox et al. (1997) and Guo et al. (2015).

Across all studied species, the genome size from the leaf tissue, floral longevity, and epidermal cell size of labellum and leaf varied considerably (Figure 1; Table 1). Specially, genome size (mean genome size ranging from 17.83 to 29.41 pg in the largest species) and floral longevity (mean floral longevity ranging from 26 to 62.13 days in the longest species) varied by up to 1.65 and 2.60 times, respectively. The epidermal cell size of labellum and leaf also varied substantially between species, with a 1.90-fold and a 2.35-fold range, respectively (Table 1).

**Table 1:** Mean values of genome size from leaf tissue (*n* = 3), floral longevity (10–20 flowers from different plants), epidermal cell size of labellum (*n* = 6), and leaf (*n* = 6) from 13 *Paphiopedilum* species.

**Species**	**1C genome size (pg)**	**Floral longevity**	**Labellum epidermal cell size**	**Leaf epidermal cell size**
		**(days)**	**(mm^**2**^ × 10^**−3**^)**	**(mm^**2**^ × 10^**−3**^)**
*P*. *appletonianum* (Gower) Rolfe	24.84 ± 0.13	53.47 ± 1.31	5.98 ± 0.32	7.70 ± 0.73
*P*. *areeanum* Gruss	21.37 ± 0.14	38.00 ± 0.33	6.47 ± 0.33	4.50 ± 0.05
*P*. *armeniacum* S. C. Chen and F. Y. Liu	18.67 ± 0.03	34.00 ± 0.63	6.05 ± 0.38	5.56 ± 0.04
*P*. *charlesworthii* (Rolfe) Pfitzer	21.48 ± 0.02	26.00 ± 0.74	6.55 ±0.46	3.72 ± 0.05
*P*. *dianthum* Tang and F. T. Wang	34.17 ± 0.07	62.13 ± 1.10	5.62 ±0.11	6.58 ± 0.06
*P*. *gratrixianum* Rolfe	21.17 ± 0.05	41.00 ± 0.56	7.46 ±0.31	4.27 ± 0.10
*P*. *henryanum* Braem	20.08 ± 0.08	32.07 ± 0.66	8.33 ±0.41	3.52 ± 0.15
*P*. *hirsutissimum* (Lindley ex Hooker) Stein	20.34 ± 0.16	37.53 ± 0.51	9.26 ±1.48	4.28 ± 0.13
*P*. *malipoense* S. C. Chen and Z. H. Tsi	22.54 ± 0.09	55.33 ± 1.09	6.64 ±0.18	7.84 ± 0.04
*P*. *micranthum* Tang and F. T. Wang	19.82 ± 0.02	28.45 ± 0.55	7.89 ±0.48	6.38 ± 0.11
*P*. *tigrinum* Koop. and N. Haseg	21.66 ± 0.13	37.27 ± 0.67	6.98 ±0.06	5.00 ± 0.05
*P*. *villosum* (Lindley) Stein	21.22 ± 0.13	35.50 ± 0.81	7.94 ±0.17	4.77 ± 0.17
*P*. *wardii* Summerh	30.96 ± 0.21	57.60 ± 0.49	4.88 ±0.16	8.26 ± 0.04

*Values shown are mean ± SE*.

**Figure 2:**
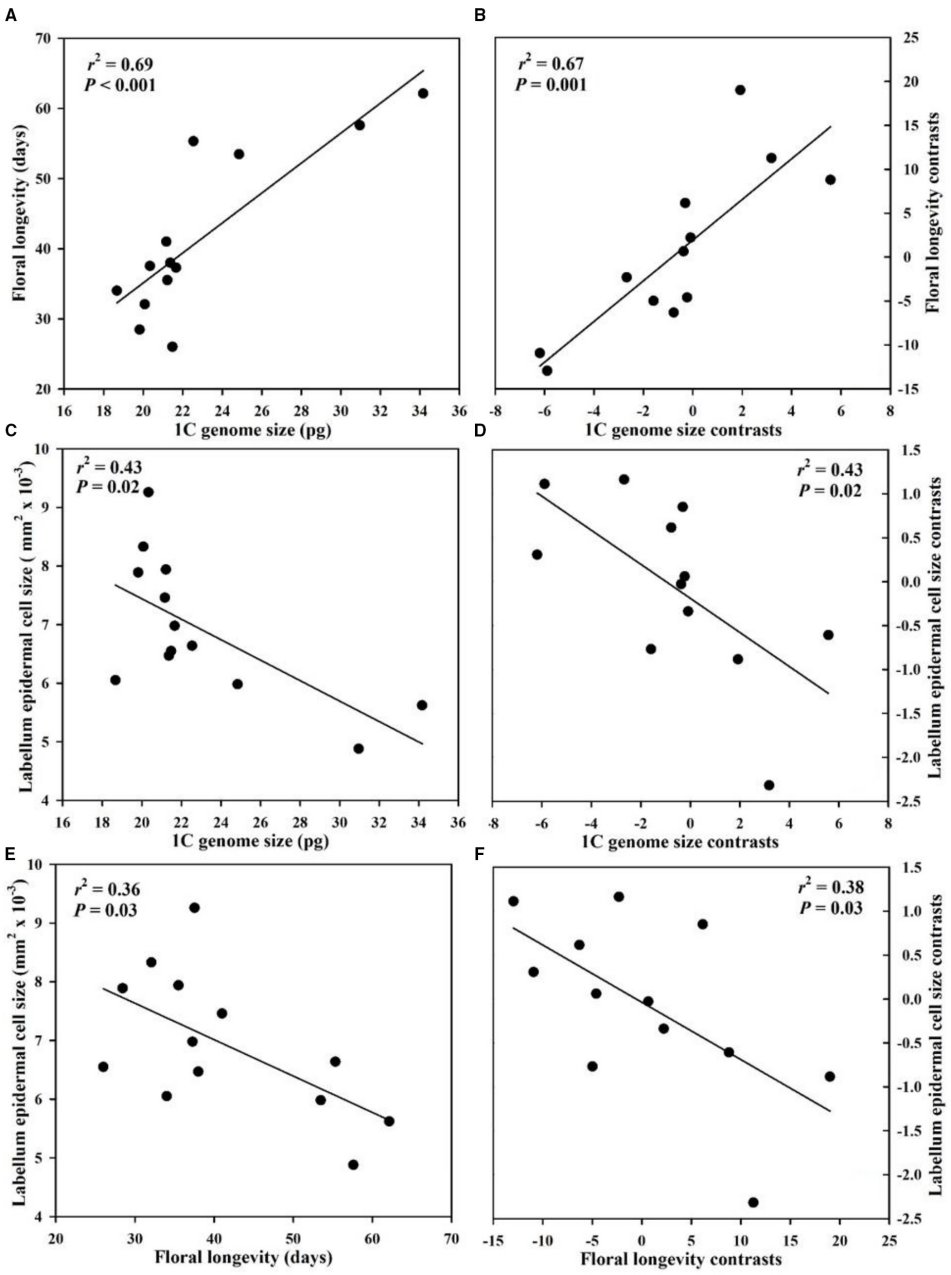
Correlations among genome size, floral longevity, and labellum epidermal cell size from 13 *Paphiopedilum* species. **(A,C,E)** Pearson's regressions; **(B,D,F)** phylogenetically independent contrast correlations.

**Figure 3:**
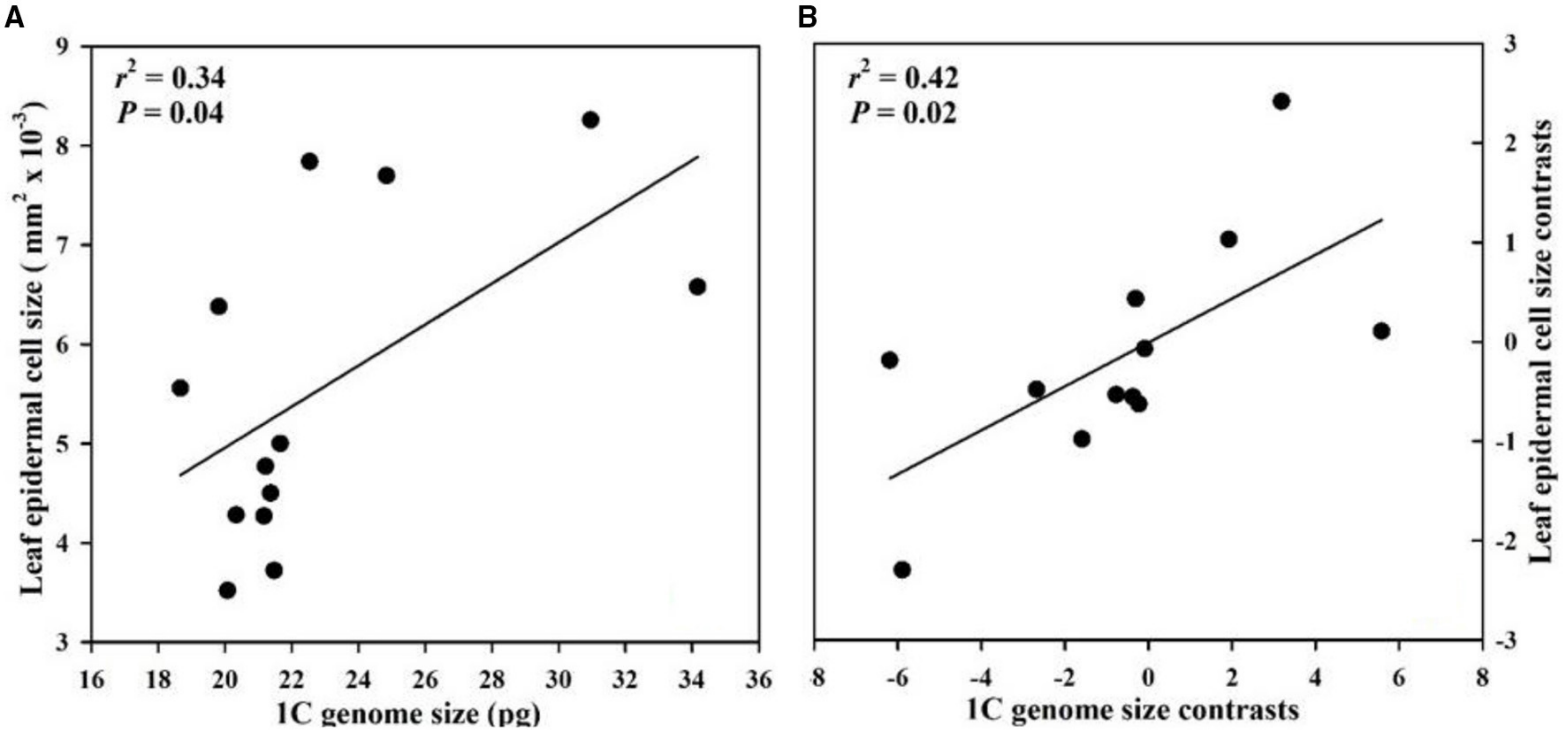
Correlations between genome size and leaf epidermal cell size from 13 *Paphiopedilum* species. **(A)** Pearson's regressions; **(B)** phylogenetically independent contrast correlations.

Genome size had a significant positive correlation with floral longevity among all species whether or not phylogeny was considered (Figure 2). The epidermal cell size in labellum had a significant negative correlation with genome size and floral longevity even after phylogeny was considered (Figure 2). In addition, genome size was positively correlated with leaf epidermal cell size in both non-phylogenetic and phylogenetic analyses (Figure 3).

## Discussion

To determine whether floral longevity was driven by genome size and cell size, we obtained data for genome size, floral longevity, and the epidermal cell size of flower and leaf for 13 *Paphiopedilum* species. We first tested whether floral longevity is associated with the sizes of genomes and epidermal cells. Our results showed that coordinated changes in genome size and cell size to potentially constraint floral longevity in flower (labellum) and leaf of *Paphiopedilum*. Our results provide new insights into the significance of genome size variation in maintaining floral longevity and evolution of floral traits in orchid plants.

Variation in genome size has aroused studies for seeking evidences as to its phenotypic consequences in plants and animals (Chung et al., 1998; Monaghan and Metcalfe, 2000; Morgan and Westoby, 2005; Beaulieu et al., 2006, 2007; Faizulah et al., 2021; Théroux-Rancourt et al., 2021). Genome size may be closely linked to cell size in biological kingdoms (Cavalier-Smith, 2005). The results may indirectly and/or directly link with whole-organism fitness values (Jockusch, 1997). Longevity is a biological trait with important fitness consequences (Monaghan and Metcalfe, 2000). Differences of longevity between species are likely to be linked to divergences in genome sizes and tissue maintenances (Kirkwood, 1985; Monaghan and Metcalfe, 2000). Our results showed that floral longevity was evolutionarily correlated with genome size and floral epidermal cell size (labellum). Species with longer floral longevity had a large genome and a small labellum cell. Our findings implied that the sizes of genome and cell played an important role in regulating floral longevity. The correlation between genome size and floral longevity that we have shown is very interesting. Whether it may be a causal link and what mechanisms might underlie it remain to be further studied. Endopolyploidy is regarded as the simultaneous existence of multiple ploidy nuclei in adjacent somatic cells of the same individual, tissue, or organ, and it is caused by normal DNA replication without cell division (Barlow, 1978). Recent studies showed that endopolyploidization commonly appeared, and levels from different tissues were different in *Orchidaceae* (Yang and Loh, 2004; Teixeira da Silva et al., 2014; Bateman et al., 2018; Li et al., 2020). For example, in *Ophyrys*, the endoreplication was frequent and (Bateman et al., 2018), while there was no evidence of endoreplication in *Dactylorhiza* (Bateman et al., 2018). In *Spathoglottis plicata*, the pedicel at the development stage showed the high level of endoploidy, but no endoploidy was found in calyx, petals, and ovary (Yang and Loh, 2004). However, the polyploidy from floral tissues of *Paphiopedilum delenatii* Guillaumin has not been demonstrated (Teixeira da Silva et al., 2014). In our study, we tried to avoid the interference of internal polyploidy by selecting young leaves. Moreover, we have measured genome sizes from labellum and root tissues, and our results showed that genome sizes from leaves and from other tissues were consistent (Supplementary Figure 3).

Floral longevity can influence the visiting frequency of pollinators, thus affecting plant reproductive rate. Meanwhile, the length of floral longevity is likely to reflect a trade-off between maintenance costs and plant fitness consequences (Kerner von Marilaum, 1895). Therefore, a trade-off should exist between investment and return in flowering plants. Time taken for DNA synthesis, and developmental duration in organs, is longer with larger genomes (Finch, 1990; Monaghan and Metcalfe, 2000; Feng et al., 2021). Within the angiosperms, the genome size of the orchids is relatively large (Leitch et al., 2009), and the reason for this is not clear. But a significant negative correlation between metabolic rate (photosynthetic rate, *A*_mass_) and genome size was found (Beaulieu et al., 2007), and the sizes of genomes and cells constraint photosynthetic rate (Roddy et al., 2020). The large genome is thought to be correlated with the low metabolic rate (Beaulieu et al., 2007).

The floral longevity in orchids is generally much longer than other flowering plant. Thus, large genome size would be possibly correlated with floral longevity. Probably, exceeding a certain value, increases of genome size may need to pay a phenotypic cost in biological function. The cell division duration in organs may be an important life history traits (e.g., floral longevity), and *Paphiopedilum dianthum* with longer floral longevity has longer duration of floral bud differentiation than that of *P*. *micrantum* and *P*. *henryanum* with shorter floral longevity (Feng et al., 2021). In this study, the strong positive correlation is between floral longevity and genome size in 13 *Paphiopedilum* species. This result contradicts previous studies showing insignificant relationship between leaf longevity and genome size (Morgan and Westoby, 2005), which suggests that flower and leaf traits *of Paphiopedilum* have evolved independently, and this possible explanation is that different selective pressures flower and leaf experienced and they acted as different functions (Roddy et al., 2013; Zhang et al., 2017).

In this study, genome size was a strong predictor of leaf epidermal cell size whether or not phylogenetic relatedness of species was considered. Genome size constrains cell size in leaves, so that cell size varies widely as cell grow and differentiate to influence its structure and function in the context of various factors, and then define phenotype (Bennett, 1987; Franks et al., 2012; Simova and Herben, 2012; Théroux-Rancourt et al., 2021), which potentially affect rates of leaf water loss (transpiration) and photosynthetic rate (Beaulieu et al., 2007; Théroux-Rancourt et al., 2021). In our study, we found that the correlation between genome size and cell size from flowers and leaves was different, implying that selective pressures they experienced may be different due to their structures and functions.

## Conclusion

We uncovered significant evolutionary correlations between floral longevity and sizes of genomes and cells in *Paphiopedilum* using a phylogenetic comparative method. These relationships between the sizes of genomes and cell and floral longevity perhaps represent a genotype and phenotype selection relationships. In addition, our results showed that genome size is a strong predictor of cell size. These findings provide novels insights into floral physiological and developmental traits and genome sizes of evolutionary correlations among flowering plant species. Therefore, sampling more species with different genome sizes in orchids and/or angiosperms should be necessary. Further investigation should pay close attention to test for a more direct influence of genome size on floral functional traits. Besides, answering the primary questions needs a continued effort to combine floral functional trait studies with the evolution of plant genome size studies. The endeavor of joining results is critical to reveal evolutionary relationships between genome sizes with floral functional traits in flowering plants.

## Data Availability Statement

The raw data supporting the conclusions of this article will be made available by the authors, without undue reservation.

## Author Contributions

F-PZ and S-BZ designed the study and wrote and revised the manuscript. F-PZ collected the samples and data, carried out the experiments, and analyzed the data. All authors contributed to the article and approved the submitted version.

## Funding

This study was supported by the National Natural Science Foundation of China (31960224), the Young Top Talents of the Ten Thousand Talents Plan in Yunnan Province (YNWR-QNBJ-2018-337), the Project for Innovation Team of Yunnan Province (202105AE160012), the Yunnan Provincial Science and Technology Department-Applied Basic Research Joint Special Funds of Yunnan University of Chinese Medicine (202001AZ070001-041).

## Conflict of Interest

The authors declare that the research was conducted in the absence of any commercial or financial relationships that could be construed as a potential conflict of interest.

## Publisher's Note

All claims expressed in this article are solely those of the authors and do not necessarily represent those of their affiliated organizations, or those of the publisher, the editors and the reviewers. Any product that may be evaluated in this article, or claim that may be made by its manufacturer, is not guaranteed or endorsed by the publisher.
