# Supplementary material for: Genome Size and Labellum Epidermal Cell Size Are Evolutionarily Correlated With Floral Longevity in Paphiopedilum Species
Source: Front Plant Sci. 2021 Dec 16;12:793516. doi: 10.3389/fpls.2021.793516 (PMC8716874; doi:10.3389/fpls.2021.793516)
Supplement: Supplementary file 1 [file Data_Sheet_1.pdf]

Genome Size and Labellum Epidermal Cell Size Are Evolutionarily

Supporting information

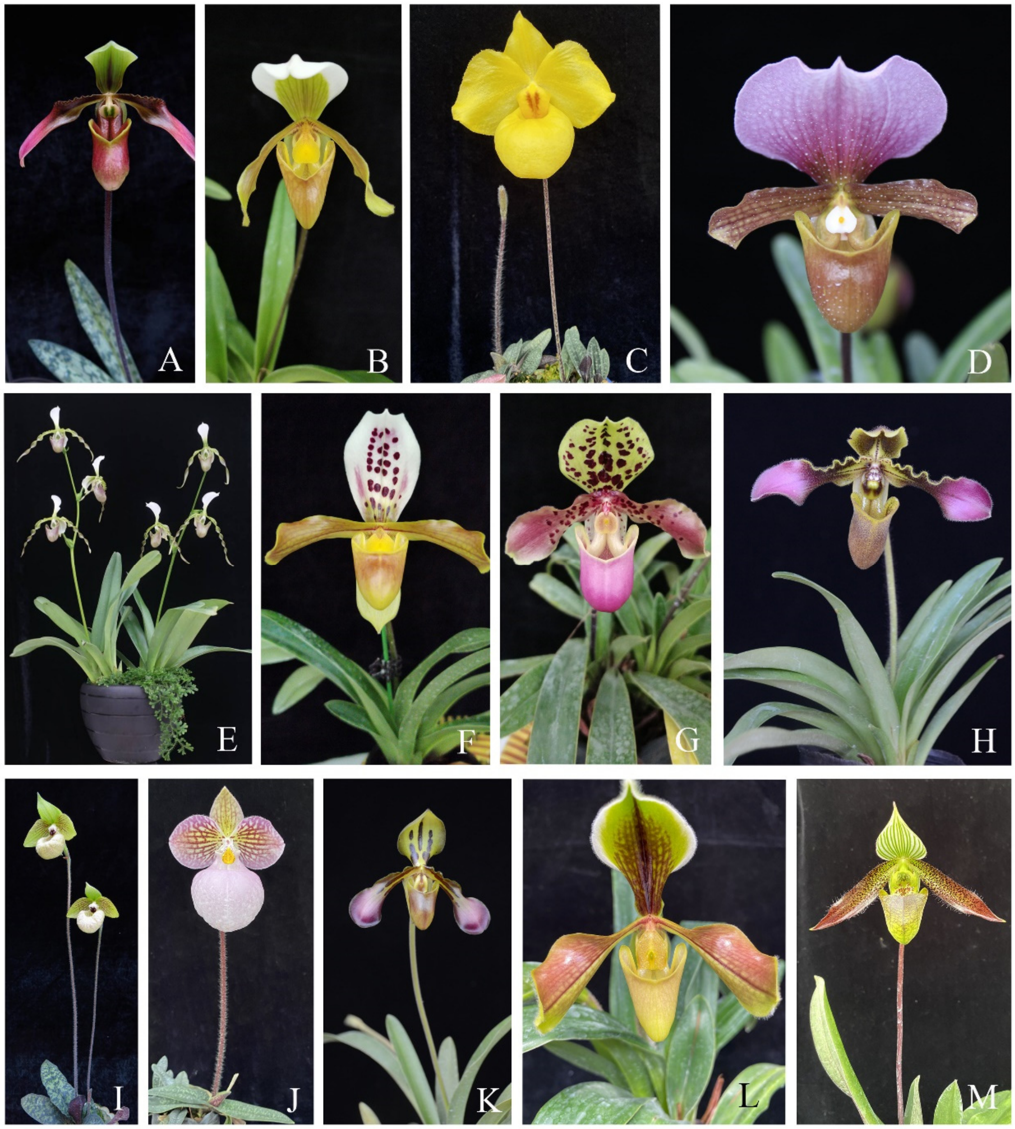

FIGURE S1 | *Paphiopedilum* species studied in this study. **(A)** *P. appletonianum*; **(B)** *P. areeanum*; **(C)** *P. armeniacum*; **(D)** *P. charlesworthii*; **(E)** *P. dianthum*; **(F)** *P. gratrixianum*; **(G)** *P. henryanum*; **(H)** *P. hirsutissimum*; **(I)** *P. malipoense*; **(J)** *P. micranthum*; **(K)** *P. tigrinum*; **(L)** *P. villosum*; **(M)** *P. wardii*

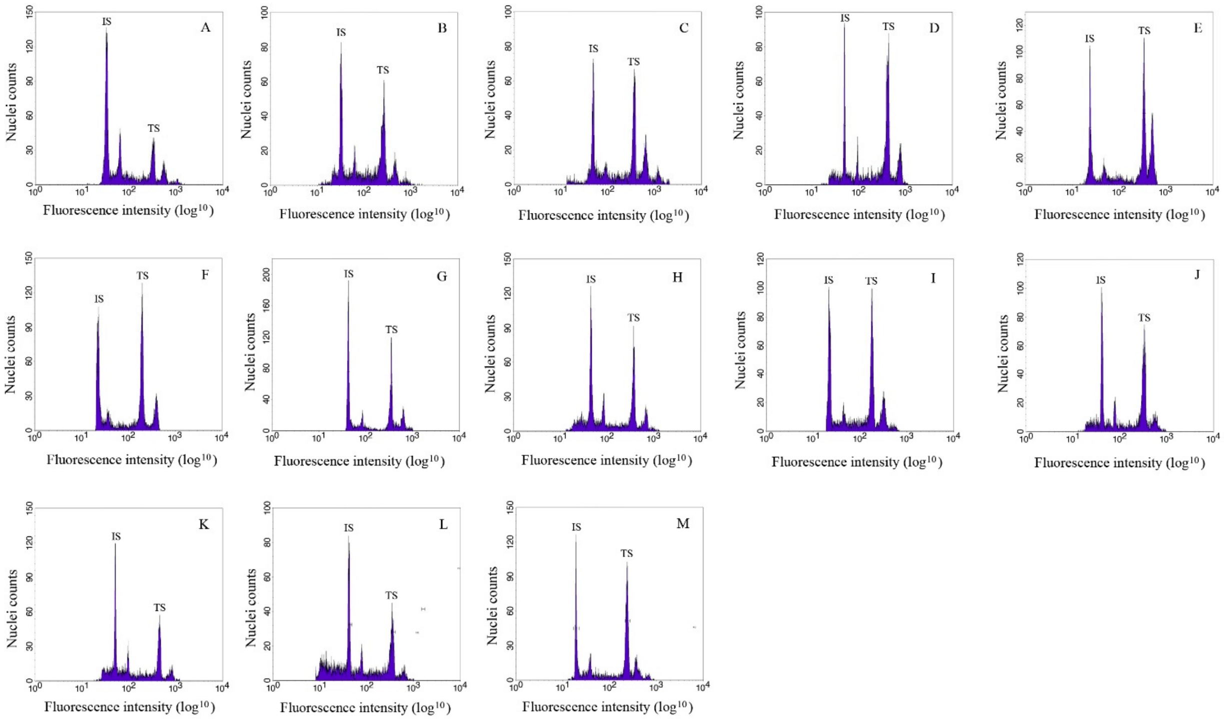

FIGURE S2 | Fluorescence intensity distributions (genome size) generated by flow cytomety from leaf tissue of *Paphiopedilum* species studied in this study. IS, internal standart (*Zea mays* L.); TS, the tested sample. The coefficient of variation values (CV) were less 5%. **(A)** *P. appletonianum*; **(B)** *P. areeanum*; **(C)** *P. armeniacum*; **(D)** *P. charlesworthii*; **(E)** *P. dianthum*; **(F)** *P. gratrixianum*; **(G)** *P. henryanum*; **(H)** *P. hirsutissimum*; **(I)** *P. malipoense*; **(J)** *P. micranthum*; **(K)** *P. tigrinum*; **(L)** *P. villosum*; **(M)** *P. wardii*

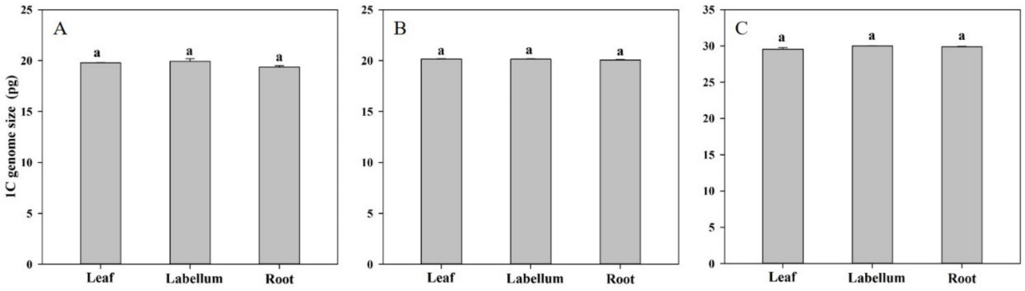

FIGURE S3丨 Means of genome sizes from leaf and labellum, and root tissues of *Paphiopedilum villosum* (A), *P. gratrixianum* (B) and *P. wardii* (C). Different letter indicate significant differences in genome size among tissues (posthoc Tukey's test, $P < 0.05$).

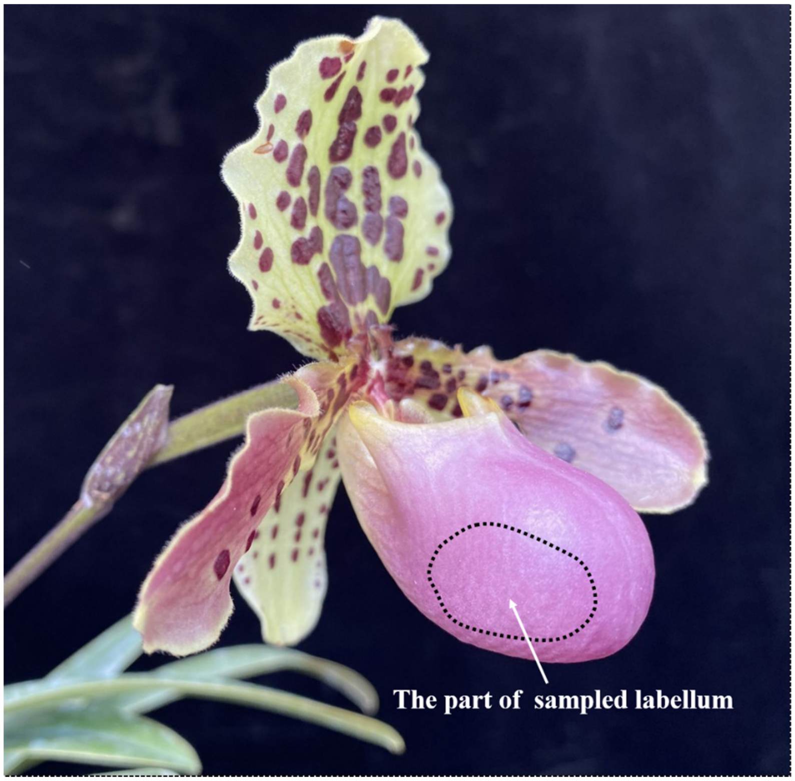

FIGURE S4 | The part of sampled labellum of cell and genome sizes in *Paphiopedilum* species (*P. henryanum*) studied in this study.
